# Rectal Surgery

**Published:** 1894-04-07

**Authors:** 


### PROGRESS IN SURGERY.

### RECTAL SURGERY.

*Hæmorrhoids.*—In spite of being a common disease and the cause of a vast amount of suffering, there is no general consensus of opinion as to the best method of treatment where operative interference is necessary; for instance, while Mr. Whitehead's operation is highly thought of in England and on the Continent, Dr. Kelsey[1] mentions it only to condemn it. So with the injection of carbolic acid into the pile, the same authority thinks the disadvantages outweigh the advantages. Mr. Treves,[2] in an excellent lecture, discusses the blood supply of the rectum, and points out that it is the superior hæmorrhoidal vein that has to do primarily with hæmorrhoids. He shows how the vessels round the anus end in some half-dozen veins which run up the bowel in the submucous tissue, and at about three inches from the anus go through holes in the muscular coat. He gives the following anatomical reasons for piles: (1) Blood is returned against gravity; (2) the veins have no valves; (3) the veins pass through muscular tissue; (4) they are affected by every form of portal obstruction; (5) they are influenced by the erect posture either sitting or standing; (6) there is an extraordinary lack of support to the veins; (7) the mechanical effect of defæcation has to be regarded. After grouping piles into (i) the external pile covered with skin, (ii) the internal pile covered with mucous membrane, and (iii) the marginal pile covered with both, and considering the external pile before, during, and after thrombosis, he passes on to the treatment of internal piles. Where an operation is necessary he recommends ligature or Whitehead's operation, and in the after-treatment to support the perinæum. Dr. Clifford Allbutt[3] in many cases of piles, fissure and the like, where there is spasm of the sphincter, and certain irritations of the rectum due to causes of a curable kind, advocates forcible dilatation of the sphincter "by the insertion of two thumbs of the operator into the orifice and the forcible separation of them until the muscle is felt to creak and give under the strain." One dilatation should be sufficient. If incontinence followed, Dr. Allbutt has not found it last more than a day or two.

P. Reclus[4] practises dilatation by means of Trélat's two-bladed speculum, obtaining anæsthesia of the part with cocaine. In sixty cases there was no incontinence after, and only one relapse.

Paul Sendler,[5] of Magdeburg, confirms the advantages of Whitehead's and Lange's method, and publishes an account of a similar method he has practised for some years. He says the patient, having been carefully prepared, is placed in the lithotomy position, and the pile drawn down, a cut is made through the skin and the pile dissected up from the loose cellular tissue and removed, the cut extending into the healthy mucous membrane and the sphincter carefully saved, after which the mucous membrane is accurately united to the outer skin. Should there be a ring of piles or an extended mass, he proceeds gradually, and stitches after removal of each pile. The average length of cure was fifteen days, the shortest four, the longest twenty-one. The functions of the sphincter were always after a short time in a normal state.

Dr. Kelsey[6] prefers the clamp and cautery. In ninety cases so treated there was no case of hæmorrhage, stricture, or other accident, and no case of recurrence. The average length of time in bed was forty-eight hours. A laxative is given at the end of that time. No after-dressing is used, except a pad and a T bandage for an hour or two.

Mr. R. Jones,[7] of Liverpool, recommends that instead of using the cautery the cut edge be sewn with a continuous catgut suture.

*Ulcers.*—Dr. J. M. Matthews,[8] of Louisville, classifies ulcers of the rectum under four heads—benign, malignant, tuberculous, and syphilitic; does not think the first-named so common as supposed; only when defæcation was interfered with was any danger to be feared. The lumen of the rectum was not narrowed by ordinary causes, and he did not believe that such causes as pregnancy, dysentery, &c., were great factors in producing ulceration of the part.

According to Hartmann,[9] tuberculous ulceration is much more common in the male sex; it commences insidiously, and is first recognised by slight pain during defæcation. It is very slow in growth, and in nearly half the cases the inguinal glands enlarge. He recommends ablation with the cautery where possible.

For *malignant growths*, Dr. Walker[10] (Detroit) writes in favour of Kraske's operation, "that of making an incision down through the second sacral vertebra to the anus to the left of the coccyx, and rapidly stripping off the muscular and ligamentous attachments to the coccyx, and cutting them away with the bone forceps, together with as much of the sacrum as was necessary to expose the growth." To approximate the cut ends of the rectum he used a large button, two inches in diameter, and so prevented the infiltration of fæcal matter. Dr. Kelsey[11] has abandoned the old perineal incision, and performs Kraske's operation, or a modification, "because even in diseases sufficiently near to the anus to be reached in this way a short dorsal incision, with preliminary removal of the coccyx, gives a much better field for removal of the lower end of the gut."

After two cases of resection of the rectum for carcinoma Gersung[12] restored the function of the sphincter. Finding it possible to draw down the bowel to meet the skin incision, he rotated the rectum in its long axis with a pair of forceps on either side until the index finger could be introduced only with difficulty, and stitched the bowel to the skin incision. Before the patients left the hospital examination showed that the funnel-shaped contraction had changed to an annular constriction acting as a normal sphincter. To obtain a new sphincter Dr. Willems[13] believes a good result might be obtained by fixing the cut edges of the rectum to a slit made in the gluteusmaximus by forcible separation of its fibres. He has practised it at present only on the cadaver. He makes an incision through the skin about two inches long over the ischial tuberosity in an oblique direction upwards and outwards. A slit is next formed in the muscle about a finger's breadth above its lower margin by separating its bundles by means of a director or closed dissecting forceps. The extremity of the gut is finally drawn through this opening and stitched to the edges of the wound. Where a considerable portion of rectum has been removed he modifies the incision to insert the gut at a higher part of the muscle.

[1] Diseases of the Rectum and Anus, 4th Edit., New York. [2] Clin. Journal, Jan. 20, 1893. [3] Clin. Journal, Jan. 24, 1894. [4] Gaz. des Hôpitaux, 1893, No. 35. [5] Centralbl. f. Chir., No. 34, 1893. [6] New York Med. J., Dec. 16, 1893. [7] Ed. Med. J., Feb., 1894. [8] New York Med. J., Nov. 4, 1893. [9] Rev. de Chir., Jan., 1894; Ex. B. M. J., Feb. 10, 1894. [10] New York Med. J., Nov. 4, 1893. [11] New York Med. J., Dec. 16, 1893. [12] Centralbl. f. Chir., 1893, No. 26; Amer. J. Med. Sci., Nov., 1893. [13] Centralbl. f. Chir.; Med. Rec., Nov., 1893.

